# Evaluation of the Bronchorelaxant, Genotoxic, and Antigenotoxic Effects of *Cassia alata* L.

**DOI:** 10.1155/2013/162651

**Published:** 2013-04-23

**Authors:** M. Ouédraogo, F. L. Da, A. Fabré, K. Konaté, C. I. Dibala, H. Carreyre, S. Thibaudeau, J.-M. Coustard, C. Vandebrouck, J. Bescond, R. G. Belemtougri

**Affiliations:** ^1^Laboratoire de Physiologie Animale, UFR-SVT, Université de Ouagadougou, 09 BP 848, Ouagadougou 09, Burkina Faso; ^2^Laboratoire de Biochimie Alimentaire, Enzymologie, Biotechnologie Industrielle et Bioinformatique (BAEBIB), UFR/SVT, Université de Ouagadougou, 09 BP 848, Ouagadougou 09, Burkina Faso; ^3^Laboratoire de Biochimie et Chimie Appliquées (LABIOCA), UFR/SVT, Université de Ouagadougou, 09 BP 848, Ouagadougou 09, Burkina Faso; ^4^Institut de Chimie des Milieux et Matériaux de Poitiers (IC2MP), UMR 7285 CNRS, Université de Poitiers-4, rue Michel Brunet (Bât B27), 86022 Poitiers Cedex, France; ^5^Institut de Physiologie et de Biologie Cellulaires IPBC, FRE 3511 CNRS, Université de Poitiers, Bât. B36-Pôle Biologie Santé 1, rue Georges Bonnet, BP 633, 86022 Poitiers Cedex, France

## Abstract

Aqueous-ethanolic extract of *Cassia alata* (AECal) and its derived fractions obtained through liquid-liquid fractionation were evaluated for their bronchorelaxant, genotoxic, and antigenotoxic effects. Contractile activity of rats' tracheas in the presence of tested materials, as well as its modifications with different inhibitors and blockers, was isometrically recorded. The antigenotoxic potential of AECal was evaluated on cyclophosphamide- (CP-) induced genotoxicity in the rat. Animals were pretreated with the extract, then liver comet assay was performed. AECal and its chloroformic fractions (CF-AECal) relaxed the contraction induced by Ach, but both were significantly less potent in inhibiting contraction induced by KCl (30 mM; 80 mM). Propranolol, indomethacin, L-NAME, methylene blue, and glibenclamide did not modify the relaxant effect of CF-AECal. TEA altered the response of trachea to CF-AECal. CF-AECal caused a rightward shift without affecting the *E*_max_ in cumulative concentration-response curves of Ach only at low concentrations. In animals pretreated with the extract, the percentage of CP-induced DNA damage decreased. Our results suggest that (1) muscarinic receptors contribute at least in part to the relaxant effects of CF-AECal; (2) CF-AECal interferes with membrane polarization; and (3) AECal is not genotoxic *in vivo* and contains chemopreventive phytoconstituents offering protection against CP-induced genotoxicity.

## 1. Introduction

Asthma is a major cause of disability, health resource utilization, and poor quality of life for those who are affected [1]. Prevalence data are lacking for many countries in Africa, but recent estimates indicate that nearly 50 million Africans currently have asthma [2]. The prevalence of the disease is the greatest (about 8% of the population) in Southern Africa [3]. It is anticipated that with continued urbanization and increasing westernization of lifestyles, the burden of asthma in Africa will continue to increase considerably in the coming decade [4].

The orthodox treatment for the management of acute attack and day to day therapy of asthma may involve the use of bronchodilators, expectorants, and corticosteroids [5]. Modern medicine is one of the largest industries in the world. However, the use of herbal remedies and traditional medicine are also rising steadily. Moreover, much of the modern scientific medicines have evolved from traditional medicine [6]. With the ability to extract such benefits from plants, our traditional system deserves an objective and critical examination. Large numbers of medicinal plants are used ethnomedically in the treatment of asthma, but there is a need to conduct pharmacological investigations to ascertain their therapeutic values. Among them, there is a shrub widely distributed in tropical countries and frequently used in Burkina Faso [7], namely, *Cassia alata* Linn. (synonym: *Senna alata*; family: Caesalpiniaceae). Antiallergic activity of *cassia alata* has been recently described by Singh et al. [8]. The plant possesses laxative, anti-inflammatory, antimutagenic, analgesic, and antimicrobial properties [9–12]. Chemical analyses of extracts from *Cassia alata *yielded constituents as phenolics, fatty acids, terpenoids, and anthraquinones [13, 14]. Infusion of leaves and flowers is used to treat asthma in Congo [15]. The torrified seeds are used as a coffee substitute and reportedly have antiasthmatic effect [16]. *Cassia alata* is used in India for the same properties [17].

The study of Pieme et al. [18] has been designed to evaluate the acute and subacute toxicities of aqueous-ethanolic extract of leaves of *Cassia alata* and provided evidence of the nontoxic effect of this extract. In another study, rats fed dried ground leaves of *C. alata* in their chow or ethanol extract added to their daily drinking water developed hepatic lesions accompanied by renal and intestinal damage [19]. Growing evidence has shown that some of the plant's secondary metabolites are toxic and/or carcinogenic, which can induce adverse effects leading to mutation and/or degenerative diseases [20]. The risk from long-term use of such remedies has not however been fully investigated, especially in terms of their potential to cause mutagenicity and carcinogenicity. Among short-term toxicity assays, the comet assay is a very sensitive test for the quantification of DNA damage. Although DNA damage as revealed by the comet assay may not necessarily result in permanent genetic damage, there is consensus about a close association of unrepaired DNA damage or error-prone repair processes, mutations, and the induction of various types of cancer [21]. In view of the above considerations, and due to the lack of information about *Cassia alata *genotoxicity, it is necessary to perform investigation on the effects of this herbal product which is often used as a therapeutic modality on genetic alterations. 

Evidence supports the potential role of antioxidant agents in cancer prevention [22]. We have therefore undertaken to investigate the antigenotoxicity of the aqueous-ethanolic extract from *Cassia alata*, given that radical scavenging antioxidant activity was reported from the aerial parts of this species [23–27].

The aim of this study was to (i) evaluate the potential bronchodilator effect of extracts from *Cassia alata*, and if any, to characterize their pharmacodynamic profile and to (ii) investigate *in vivo* the genotoxic and antigenotoxic effects of aqueous-ethanolic extract of *C. alata *leaves. 

## 2. Materials and Methods

### 2.1. Collection of Plant Material, Extraction, and Fractionation

*Cassia alata *was collected in December 2010 in Komsilga that is located 15 km from south of Ouagadougou. Botanical identification was done by Assistant Professor Amade Ouédraogo by comparison with an authentic specimen deposited at herbarium of the Department of Plant Biology and Physiology, University of Ouagadougou. Voucher specimen under the ID 15965 was deposited at the herbarium of the same department.

The powdered leaves (1 kg) were extracted by repeated maceration with 80% aqueous-ethanol solvent (5 l, 5 × 48 h) at room temperature. It was filtered through a cloth and then through a filter paper. The combined ethanol solutions were concentrated on rotary evaporator under reduced pressure, frozen, and lyophilized to dryness yielding approximately 13.75%.

A known quantity of extract (10 g) was dissolved in distilled water (75 mL). This was then introduced in separating funnel, and petroleum ether (75 mL) was added. The mixture was shaken vigorously, allowing the air to escape out regularly. It was kept for about 30 min to let the two layers separate. The upper petroleum ether layer was acquired, and the same procedure was repeated twice. All the petroleum ether layers were collected and concentrated on rotary evaporator to obtain the petroleum ether fraction (PEF-AECal). Chloroform (75 mL) was then added to the remaining layer, and the same process was repeated as with the petroleum ether. Finally, we got the chloroform fraction (CF-AECal). The ethyl acetate (EAF-AECal) and n-butanol (nBF-AECal) fractions were, respectively, acquired using the same procedure.

### 2.2. Animals

Wistar rats (180–225 g) of either sex were anaesthetized with intraperitoneal injection of chloral hydrate (2 g/kg) or diethyl ether. All animal handling and procedures strictly conformed to the Guide for the Care and Use of Laboratory Animals (National Institutes of Health, Publication no. 85-25, revised 1996).

### 2.3. Isolated Rat Trachea Experiments

#### 2.3.1. Isolated Tissue Preparation

After the rat was deeply anaesthetized with chloral hydrate, its chest was open by thoracotomy, and the trachea was quickly removed. Then, the trachea was placed into Krebs solution containing the following (in mM): 120 NaCl, 4.7 KCl, 2.5 CaCl_2_, 1.2 MgCl_2_, 1.2 KH_2_PO_4_, 15 NaHCO_3_, 11.1 D-glucose, and pH 7.4. The trachea, cleaned from connective tissues, was cut into rings of 3-4 mm length, which were suspended between a fixed clamp and a platinum hook attached to a force transducer (FT) (Model FT03, Grass Instruments, Quincy, MA, USA), then was placed in a water-jacketed 10 mL organ bath containing oxygenated Krebs solution [28]. Tension was recorded by MacLab/8e digitizer (AD Instruments, Castle Hill, NSW, Australia), driven by PowerLab Chart* v.*4.2 software (AD Instruments), through an ETH-400 bridge amplifier (CB Sciences, Dover, NH, USA). The signal was recorded and stored in binary files in a personal computer. The rings were rinsed three times and equilibrated in Krebs solution for 1 H, and then the basal tension was monitored and adjusted to 2 g. 

#### 2.3.2. Protocols

After the equilibration period, the relaxant actions for cumulative addition of extract and fractions against acetylcholine (10 *μ*M) or KCl (30 mM; 80 mM) induced contractions of tracheal rings were performed. The high K^+^ depolarization was acquired by replacing NaCl with equimolar KCl, in order to maintain osmolarity [29]. 

To examine the possible participation of cholinergic stimulation, ACh-induced contractions were carried out in tracheal rings before and after preincubation with AECal, CF-AECal, or atropine (a competitive nonselective muscarinic receptor antagonist). To characterise the possible contribution of epithelium-derived nitric oxide/cyclic guanosine monophosphate (ENDO/cGMP) relaxant pathway, relaxations to CF-AECal against ACh-induced contraction were performed in tracheal rings preincubated with L-NAME (a nonspecific nitric oxide synthase (NOS) inhibitor (100 *μ*M)) or methylene blue (a soluble guanylyl cyclase inhibitor (10 *μ*M)). To investigate possible interactions between CF-AECal and prostaglandins, tissues were incubated with indomethacin (10 *μ*M, cyclooxygenases inhibitor), then CF-AECal was added at different concentrations, and cumulative concentration-response curves were obtained. In order to know the role of K^+^ channels in CF-AECal-induced relaxation, tracheal rings were incubated for 15 min with K^+^ channel blockers (glibenclamide, 10 *μ*M, or tetraethylammonium (TEA, 5 mM)), then CF-AECal was added at different concentrations, and cumulative concentration-response curves were obtained. To investigate the possible participation of sympathetic pathways (*β*_2_-adrenergic receptor agonistic activity), relaxation to CF-AECal was done in trachea rings preincubated with propranolol (1 *μ*M), a nonselective *β*-adrenergic receptor blocker. 

### 2.4. Genotoxicity and Antigenotoxicity Evaluations

#### 2.4.1. Animals, Treatments, and Experimental Design

Twenty-four male rats were randomly distributed into eight groups with three animals each. The negative control group was given solvent by oral gavage; the positive control group was given a single intraperitoneal injection of the equivalent of 40 mg/kg b·w of cyclophosphamide dissolved in distilled water; the treatment group was given 250, 500, or 1000 mg/kg b·w of aqueous-ethanolic extract of *C. alata *each day for 7 days by oral gavage; and the experimental group was given the same treatment as the treatment group except that, on the seventh day, the rats also received the same treatment as the positive control group. 

#### 2.4.2. Single Cell Gel Electrophoresis

The comet assay (alkali method) was performed following the procedure of Singh et al. [30] with a slight modification. Microscope slides, frosted on one end, were dipped briefly into 1.5% hot (60°C) normal melting agarose prepared in phosphate-buffered saline (PBS). For controls and treated animals, a part of the large lobe of the liver was removed and placed onto a small Petri dish with ice-cold buffer (HBSS) containing 25 mM ethylenediaminetetraacetic acid (EDTA) and 10% dimethyl sulfoxide (DMSO) at pH 7.5. The liver samples were cut into smaller pieces and washed to remove as much blood as possible. Then, a fresh mincing solution was added, and the liver samples were minced into finer pieces. Subsequently, 5 *μ*L of the cell suspension, mixed with 75 *μ*L of 0.75% low melting point agarose (LMPA) at 37°C, was spread on the slide using a coverslip and then allowed to solidify at 4°C in a moist box. After removal of the coverslip, the slides were immersed in freshly prepared cold (4°C) lysing solution (2.5 M NaCl, 100 mM EDTA, 10 mM Tris; pH 10; 1% Triton X-100, and 10% DMSO added just before use) for at least 1 H. After lysis, the microscope slides were placed in an electrophoresis unit, allowing DNA to unwind for 40 min, in the electrophoretic buffer consisting of 300 mM NaOH, 1 mM EDTA, and pH > 13. Subsequently, the DNA was electrophoresed. Electrophoresis was conducted at temperature of 4°C for 30 min at 300 mA and 25 V. The slides were then neutralized with 0.4 M Tris, pH 7.5, stained with propidium iodide, and covered with cover slips. To prevent an additional damage, all the steps described above were conducted under dimmed light or in the dark.

#### 2.4.3. Comet Analysis

The following study design has been applied: 50 cells scored per slide, replicate slides from each experimental unit, and 3 animals per group. The slides were observed at 400x magnification, in a Nikon Labophot fluorescence microscope attached to a Sony 3CCD video camera and connected to a personal computer-based image acquisition system. Images were analysed using *CometScore* software (version 1.5 of TriTek Corporation, free download from http://www.autocomet.com/). DNA damage was quantified as percentage of DNA in tail (% tail DNA) [31].

### 2.5. Drugs

Acetylcholine chloride, atropine, propranolol hydrochloride, isoprenaline, N_*ω*_-nitro-L-arginine methyl ester hydrochloride (L-NAME), indomethacin, glibenclamide, tetraethylammonium, cyclophosphamide, Hank's Balanced Salt Solution (HBSS), ethylenediaminetetraacetic acid (EDTA), Trizma base, Triton X, and propidium iodide were purchased from Sigma Chemicals. Co., Germany. Methylene blue was from Merck (Darmstadt, Germany). All chemicals were dissolved in water except for propranolol indomethacin and glibenclamide which were dissolved in DMSO. Further dilutions were made in Krebs solution. The final DMSO concentrations did not significantly affect the results. 

### 2.6. Data Analysis

Results from bronchorelaxant effect evaluation are expressed as means ± SEM of measurements in *n*preparations from different animals, except for the EC_50_, where it is given as the geometric mean accompanied by its respective 95% confidence limit. The EC_50_ (i.e., the concentration of drug causing half-maximum relaxant response) and the pEC_50_ (the negative logarithm to base 10 of the EC_50_ of an agonist) were calculated in each concentration-response curve, by nonlinear regression analysis. Student's *t*-tests for paired and unpaired samples were used to determine the significance of differences between mean values in all control and test tissues. 

For the comet assay, data were statistically analyzed using Prism Software. Normality and homogeneity of variance were verified by Kolmogorov-Smirnov test and Bartlett's test, respectively. Analysis of variance (ANOVA) for multiple-sample comparison was applied when normality and homogeneity of variance in several distributions of investigated parameters were satisfied, followed by Tukey's *post hoc* analysis. Nonnormal data were log-transformed to attain normality, thus allowing the application of parametric statistics ANOVA [32]. 

Data were analyzed with GraphPad software (GraphPad Software Inc., San Diego, CA, USA). Differences were considered statistically significant when *P* values were <0.05 (*), <0.01 (**), and <0.001 (***).

## 3. Results

### 3.1. Bronchorelaxant Effect

#### 3.1.1. Inhibition of Basal Tone of the Trachea by Extracts

AECal and CF-AECal at lower concentrations (from 0.001 to 0.1 mg/mL) did not influence significantly the basal tone of the tissues. However, at higher concentrations (≥0.3 mg/mL and ≥1 mg/mL, resp., for CF-AECal and AECal), these substances modulated significantly the basal tone of tracheal rings (*P* < 0.05 versus paired control). 

#### 3.1.2. Effects of Extracts and Different Fractions on KCI- and Acetylcholine-Induced Contractions

**Figure 1:**
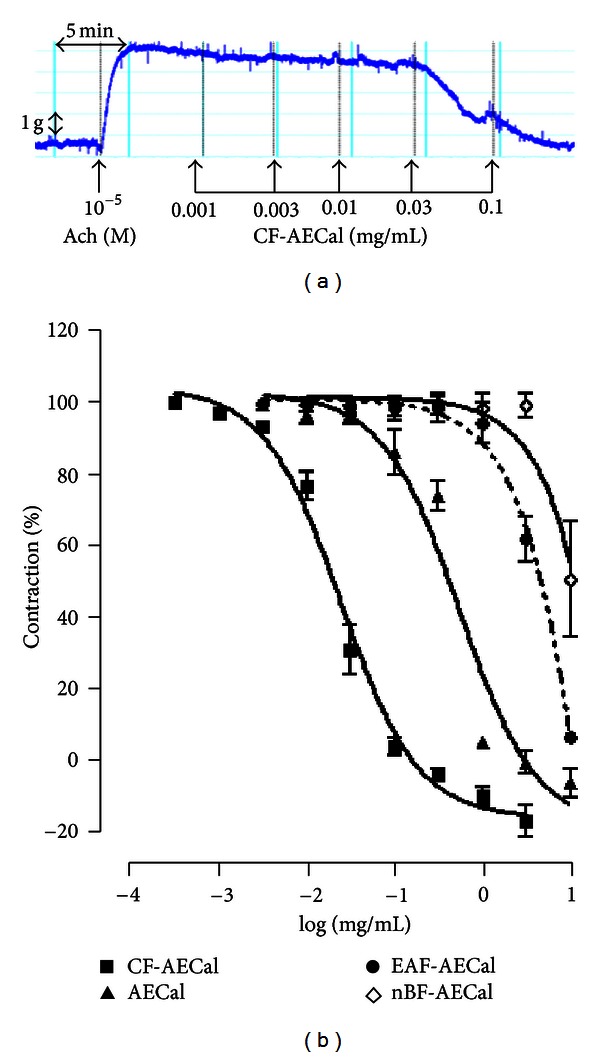
(a) Typical tracing showing the relaxant effect of CF-AECal on acetylcholine (ACh, 10^−5^ M) induced contractions in isolated rat trachea preparation. (b) Relaxing concentration-response curves of crude aqueous-ethanol extract (▲), chloroformic (■), ethyl-acetate (*⚫*), and n-butanol (◊) fractions of crude extract from *Cassia alata* on mouse tracheal rings precontracted with acetylcholine. Ordinate scale: percentage reduction in responses to ACh 10^−5^ M as appropriate. Each point is the mean derived from at least 6 experiments; vertical lines show sample SEM means values.

Addition of single concentration of acetylcholine (10^−5^ M) produced a contractile response which averaged 4.25 ± 0.68 g (*n* = 7). AECal and CF-AECal (0.001–0.1 mg/mL) produced a concentration-dependent relaxation of the contractile responses induced by acetylcholine (Figure 1(a)). Comparison of pharmacological indices (EC_50_, pEC_50_) revealed that the chloroformic fraction is tenfold more potent than the aqueous-ethanol extract. We observed that CF-AECal was the most potent fraction against the contractile responses induced by this contractant, whereas PEF-AECal, EAF-AECal, and nBF-AECal exhibited negligible effects (Figure 1(b), Table 1).

**Figure 2:**
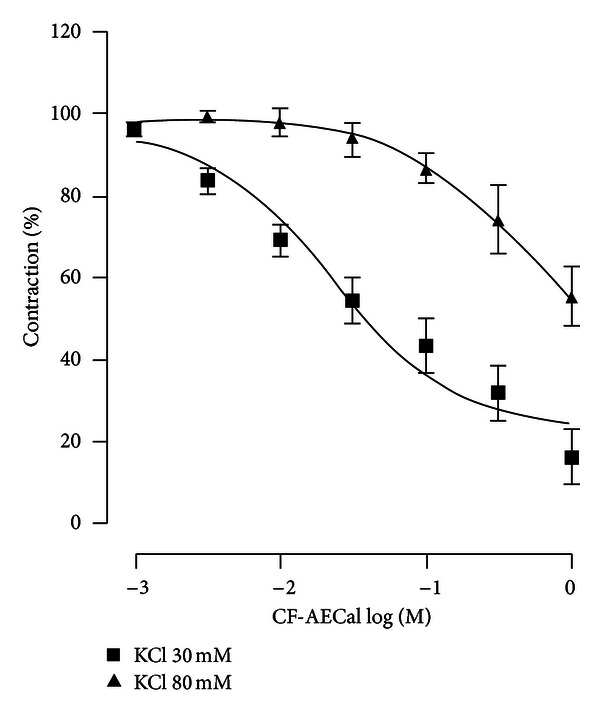
Rat isolated trachea: relaxant activity of CF-AECal against established contraction to KCI 30 mM (■) and KCI 80 mM (▲). Abscissae: −log molar concentration of CF-AECal. Data are expressed as the mean ± SEM of six experiments.

Figure 2 shows the effect of CF-AECal on various concentrations of KCl (30, 80 mM)-evoked responses in the trachea. This fraction caused an incomplete and concentration-dependent relaxation of rat's isolated trachea contracted with 30 mM KCl. The relaxation concentration-response curve to CF-AECal against KCl 80 mM-induced contraction was shifted to the right approximately tenfold compared with CF-AECal relaxant activity against KC1 30 mM. Moreover, only partial relaxation which corresponded to approximately 55.47 ± 7.42% of the 80 mM KC1-induced contraction was achieved (Figure 2, Table 2).

The EC_50_ values against the contractions induced by these two bronchoconstrictors are compared in Table 2. CF-AECal relaxed acetylcholine-evoked contractions more strongly than high K^+^-evoked contractions from 30 mM to 80 mM.

#### 3.1.3. Antagonism of Contractions Induced by Acetylcholine

**Figure 3:**
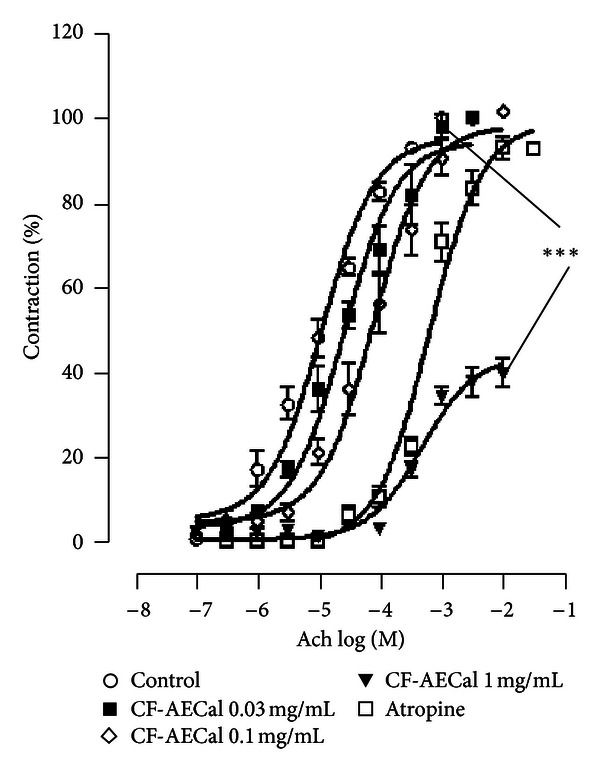
Concentration-response curves of acetylcholine (ACh) in the absence (○) and presence of 0.03 mg/mL of chloroformic fraction of *Cassia alata* (CF-AECal) (■), 0.1 mg/mL of CF-AECal (◊), 1 mg/mL of CF-AECal (*▼*), and atropine (□) in isolated rat trachea preparations. Values shown are mean ± SEM, *n* = 5–7. Significantly different from control: *P < 0.05, **P < 0.01, and ***P < 0.001.

Cumulative log concentration-response curves of acetylcholine obtained in the presence of CF-AECal and atropine (10^−7^ M) showed clear rightward shift compared to acetylcholine-response curves produced in the presence of saline (Figure 3). The shift in ACh-curves engendered by CF-AECal is small but significant and parallel at low concentrations (0.01 and 0.1 mg/mL). Pretreatment of the tissue with higher concentrations (0.3 and 1 mg/mL) caused a nonparallel shift (Figure 3). The maximum responses to acetylcholine obtained in the presence of CF-AECal at low concentrations remained unaltered. Similar results were obtained in rat isolated tracheas incubated with atropine (0.1 *μ*M). However, the efficacy of acetylcholine in the presence of CF-AECal at higher concentrations was significantly decreased comparatively to controls (*P* < 0.01) (Figure 3, Table 3).

#### 3.1.4. Effects of Propranolol, L-NAME, Indomethacin, and Methylene Blue on Relaxation Induced by CF-AECal

**Figure 4:**
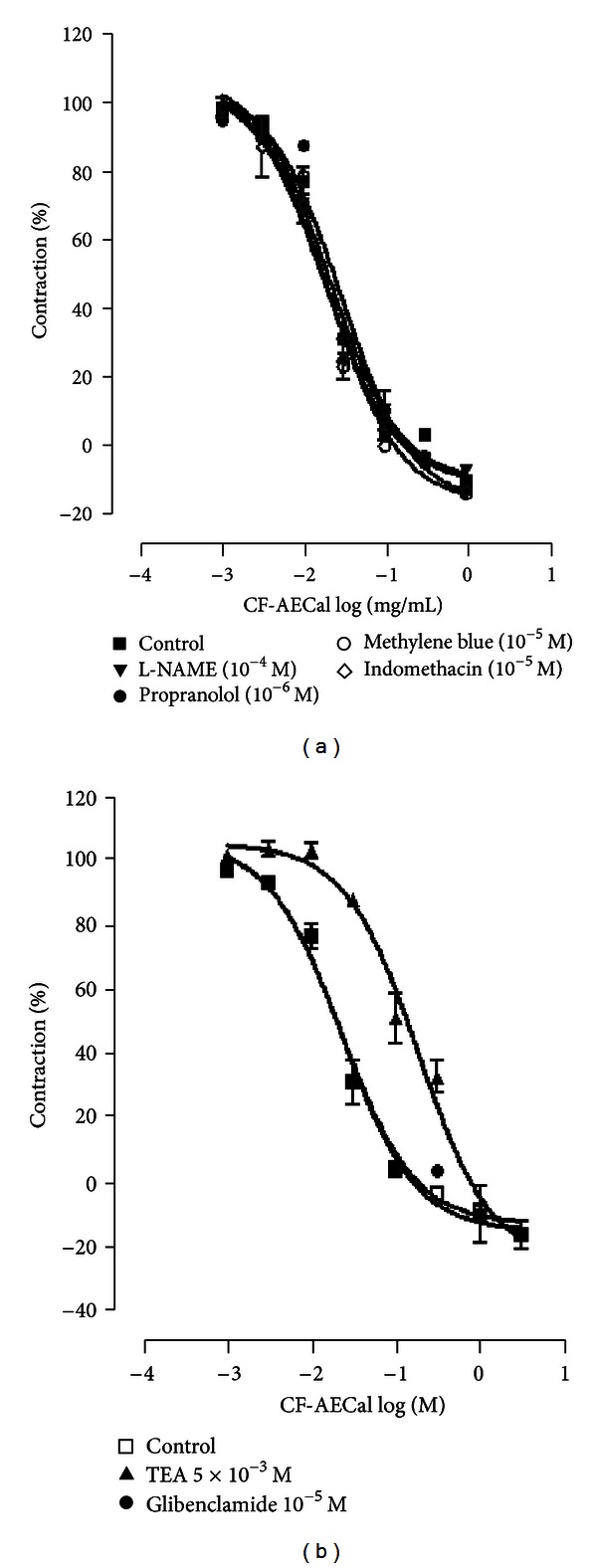
(a) Relaxing concentration-response curves of CF-AECal (■) on rat tracheal rings pretreated with L-NAME (*▼*), indomethacin (◊), methylene blue (○), and propranolol (*⚫*) and precontracted with acetylcholine (10 *μ*M). (b) Relaxing concentration-response curves of CF-AECal (□) on rat tracheal rings pretreated with glibenclamide (*⚫*) and tetraethylammonium (▲) and contracted with acetylcholine (10^−5^ M). Data are expressed as the mean ± S.E.M. of six experiments.

In order to evaluate the contribution of adrenoceptors to the relaxant action of CF-AECal, tracheal rings were firstly preincubated with propranolol (1 *μ*M), a selective *β*-adrenoceptor antagonist. Propranolol did not antagonize the relaxation induced by CF-AECal added cumulatively (Figure 4(a), Table 4).

The bronchodilator effects of CF-AECal were analyzed in tracheal chains incubated with L-NAME (100 *μ*M) or indomethacin (10 *μ*M). The incubation with L-NAME or indomethacin did not significantly affect the relaxant response to CF-AECal on acetylcholine precontracted tracheas. The relaxation elicited by CF-AECal in the presence of the guanylate cyclase inhibitor MB (100 *μ*M) was not significantly different from that produced by CF-AECal alone on ACh-induced contraction (Figure 4(a), Table 4). 

#### 3.1.5. Effects of K^+^ Channel Blockers on CF-AECal-Induced Bronchodilation in the Trachea

The possible role of K^+^ channels in CF-AECal-induced bronchodilation was analyzed using K^+^channel blockers. Addition of tetraethylammonium (5 mM), a broad-spectrum K^+^ channel blocker, produced a transient contraction on tracheal chains. TEA decreased significantly the bronchorelaxant potency of CF-AECal on acetylcholine-prestimulated tissues. An eightfold EC_50_ value decrease was recorded when tested in the presence of TEA. Glibenclamide (10 *μ*M), an ATP-sensitive K^+^ (K_ATP_) channel blocker, had no significant effect on CF-AECal-induced relaxations. The relaxant effects of CF-AECal were similar in the absence and in the presence of glibenclamide (Figure 4(b), Table 4).

### 3.2. Single Cell Gel Electrophoresis (Comet Assay)

**Figure 5:**
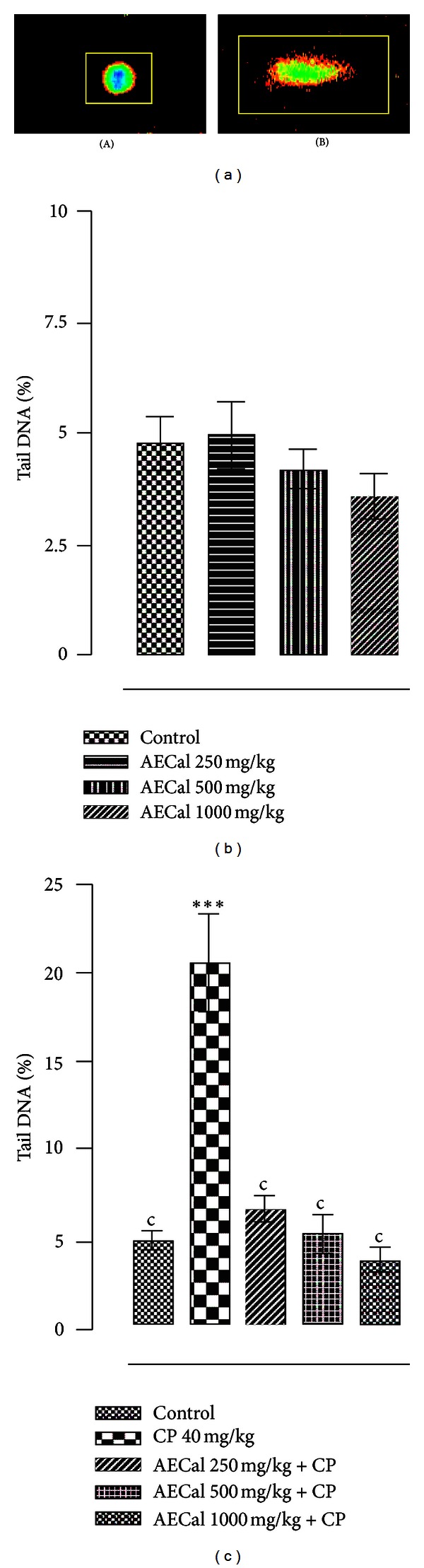
(a) Rat-liver comet assay: representative comet images from (A) treatment with AECal and (B) CP-treated cell (positive control). DNA was stained with propidium iodide; 400x magnification. Images were treated with the CometScore software to eliminate the background from the field of view before acquiring comet metrics. (b) Genotoxic effects of aqueous-ethanolic leaf extract of *Cassia alata* assessed by comet assay in rat livers cells. *P < 0.05, **P < 0.01, and ***P < 0.001 versus negative control (ANOVA followed by Tukey's test). (c) Antigenotoxic effects of aqueous-ethanolic leaf extract of *Cassia alata* assessed by comet assay in rat livers cells. *P < 0.05, **P < 0.01, and ***P < 0.001 versus negative control; ^a^ *P* < 0.05, ^b^ *P* < 0.01, and ^c^ *P* < 0.001 versus the corresponding cyclophosphamide alone (ANOVA followed by Tukey's test).

Figure 5(a)(A) shows representative undamaged DNA resulting from the exposure of rat to AECal (250–1000 mg/kg). The % tail DNA for the liver cells from extract treated rats, as compared with appropriate controls, are presented in Figure 5(b). It can be seen from this figure that AECal at the administrated concentrations did not evoke a significant effect on DNA migration. None significant difference between AECal-treated and untreated animals has been observed for all three tested doses. Statistical analysis showed that extent of DNA damage did not decreased significantly with increasing dose of AECal administration, and there were no significant differences among the treatment doses (Figure 5(b)).

As expected, cyclophosphamide, the positive control, induced a significant increase in DNA migration (*P* < 0.001) (Figures 5(a)(B) and 5(c)). It can be seen from this table that comets resulting from cyclophosphamide exposed rats liver cells contain more DNA in their tails than comets resulting from control group liver cells. The induced damage caused by the *in vivo* treatment with cyclophosphamide decreased significantly in all groups when cotreated with the crude aqueous-ethanolic extract (Figure 5(c)). AECal at the highest dose (1000 mg/kg b·w) caused an overall significant reduction of the induced DNA damage.

## 4. Discussion

The results of the present study indicate that AECal and CF-AECal relaxed or antagonized induced contractions of the rat isolated tracheal muscle preparations in a concentration-related manner, suggesting that these tested materials possess pharmacological properties as bronchorelaxants. 

The parallel rightward shift in acetylcholine response curves obtained in the presence of CF-AECal, compared to that of saline, and the nonsignificant change in the maximum responses to acetylcholine obtained in the presence of this fraction at low concentrations show possible competitive antagonistic effects of this fraction on muscarinic receptors. The nonparallel rightward shift in acetylcholine log concentration-response curves obtained in the presence of CF-AECal at higher concentrations, greater EC_50_, but lower maximum contraction effect to acetylcholine, compared to those of saline, indicates a functional antagonistic effect of this fraction [33–35].

It is unlikely that the mechanism of the bronchodilatory action of CF-AECal involves the stimulation of the *β*_2_-adrenoceptors present on bronchial smooth muscles. This hypothesis is supported by the observation that concentration of propranolol which completely abolished the bronchospasmolytic effect of isoprenaline did not affect the bronchospasmolytic action of this chloroformic fraction. Further, agents that inhibit the synthesis (L-NAME) or antagonize the action (methylene blue) of NO, a bronchodilator agent known to be produced by tissues within the respiratory system [36–38], failed to inhibit the CF-AECal effects on isolated tracheal rings. Bronchorelaxant prostaglandins do not significantly contribute to the CF-AECal-induced relaxations, as indomethacin did not affect this activity. 

The relatively nonselective potassium channel antagonist, tetraethylammonium ion (5 mM), shifted the concentration-relaxation curve for CF-AECal eightfold to the right, whereas glibenclamide, an ATP-sensitive potassium channel blocker, did not alter CF-AECal-induced bronchodilation. Imaizumi and Watanabe [39] reported that TEA probably increased the transmembrane Ca influx as a result of the potentiation of cell membrane depolarization, when electrically, mechanically, or chemically stimulated. Consequently, contraction-promoting effect of TEA may explain the eightfold decrease in the potency of CF-AECal. Sausbier et al. [40] demonstrated that BK channel plays a predominant role in the regulation of airway tone. Independently of any induced-contraction, CF-AECal decreased the tracheal smooth muscle basal tone, evoking a possible membrane hyperpolarization. Taken together, these observations attribute to the regulation of membrane polarization by CF-AECal a preponderant role.

In rat trachea, CF-AECal strongly inhibits the contractions induced by low concentrations of KCl (30 mM) as opposed to high concentrations (80 mM) of KCl. Such a pharmacological profile has been described for K^+^ channel openers [41, 42] which are able to block smooth muscle contractions induced by low K^+^ concentrations (<30 mM), but not high depolarizing K^+^ concentrations (80 mM). With high depolarizing K^+^ concentrations, potassium equilibrium potential and cell membrane potential are so close that hyperpolarization induced by K^+^ channel opening is too weak to close voltage-operated Ca^2+^ channels [43]. 

High K^+^-induced bronchoconstriction results from the depolarization of cell membrane, which causes the augmentation of calcium influx through voltage-operated calcium channels leading to the rise of intracellular calcium level [44]. A substance which can inhibit the high K^+^-induced contraction is therefore considered to be a possible calcium channels blocker. CF-AECal moderately relaxes the K^+^-induced contraction at higher concentrations suggesting the implication of a Ca^2+^ antagonist modest effect or the modulation of Ca^2+^ mobilization. 

Moreover, an increase in intracellular Ca^2+^ concentrations ([Ca^2+^]i) and the subsequent Ca^2+^-calmodulin-dependent activation of myosin light chain kinase is the main determinant of smooth muscle contraction [45]. Kaempferol and kaempferol-3-O-gentiobioside were both reported from *C. alata* leaves. Kaempferol is a potent inhibitor of myosin light chain kinase in vascular smooth muscle [46]. The bronchospasmolytic action of CF-AECal at high concentrations might be due to the presence of flavonoids, including kaempferol, as these compounds have been reported to possess myorelaxant activities. However, contribution of other constituents cannot be ignored.

The second objective of experiments designed in this study attempts to evaluate the genotoxic/antigenotoxic potential of *Cassia alata* leaves extract. Genotoxic studies are useful to identify the level of DNA damage induced by xenobiotics, as well as to give a clue about the possible clinical consequences of human exposure. 

There was no significant difference in tail DNA % (*P* > 0.05) in the rats treated with *Cassia alata* when compared with negative control. The data obtained indicate that aqueous-ethanolic extract of *Cassia alata *did not induce DNA migration in the liver cells comet assay when it is administrated in doses with ranges between 250 mg/kg and 1000 mg/kg. This absence of DNA migration indicates probably that the whole plant extract, at least under the experimental conditions examined, was not able to produce any single-strand and double-strand DNA breaks. These results are of particular relevance because this plant extract is very often used as a therapy in the ethnopharmacological context. Similar results have been reported by Akinmoladun et al. [24]. They indicated that* Cassia alata *leaves extract did not show any cytotoxic effect in wild-type strains of the yeast *Saccharomyces cerevisiae. *However, these authors observed that higher than wild-type sensitivity to *Cassia alata* was exhibited by *Saccharomyces cerevisiae *mutants with defects in DNA repair and membrane transport. Kaempferol-3-O-*β*-D-glucoside (astragalin) has been identified as the probable cause responsible for this toxicity. Nevertheless, there may be limitations to the utility of yeast as a mammal surrogate, due to differences in the molecular environment and the more complex genetic interactions in mammals. Some biological effects induced by kaempferol *in vitro*, including some toxic effects, may not be relevant *in vivo *when this flavonoid is taken orally [47]. These considerations are in line with the results from the investigations of Heidemann et al. [48]. They reported that the extracts of *Cassia senna* and aloe-emodin were genotoxic when tested with some *in vitro* test systems. Under *in vivo* conditions so far, no genotoxic effect was found. Interstrand crosslinks (ICLs) prevent separation of the DNA strands. Their presence results in less DNA migration in the comet assay [49]. In further studies, implementation of test aimed to detect extract-induced ICLs has to be performed. These experiments will be useful to confirm the nongenotoxicity of AECal or to find other explanation to the absence of DNA migration in treated groups.

DNA damage increased significantly after treating rats with cyclophosphamide, while a significant protective dose-effect was found in the presence of the extract. CP requires metabolic activation by the cytochrome P-450-dependent monooxygenase system. The main effect of cyclophosphamide is due to its metabolite phosphoramide mustard. Phosphoramide mustard forms DNA interstrand and intrastrand crosslinks [50]. Nitrogen mustards (NMs) form cyclic aziridinium rings then alkylate DNA. A second attack forms the second alkylation that results in the formation of interstrand crosslinks (ICLs) [51]. CP has the ability to generate free radicals that cause endothelial and epithelial cell damage [52]. Since CP requires metabolic activation before inducing DNA damage, AECal could have stimulated phase II enzymes and eliminated CP metabolites. Moreover, AECal could have acted as reactive species quenchers. In fact, several studies reported the free radical scavenging ability in *Cassia alata* extracts [23–26]. AECal*-*induced synthesis of antioxidant enzymes and inhibition/stimulation of specific proteins involved in DNA damage repair remain to be explored.

Isolation and characterisation of the active antioxidant compound in the methanol extracts from the aerial parts of *Cassia alata* led to identification of the flavonol named kaempferol [23]. Leaf extracts from *Cassia alata *L. fractionation yielded a new indole alkaloid, 1-(4′-hydroxyphenyl)-2,4,6-trihydroxy-indole-3-carboxylic acid, which demonstrated a strong antioxidant potential [25]. Kaempferol reduces the DNA damage induced by mutagenic compounds in the human comet assay [53]. Pretreatment of human lymphocytes with kaempferol reduced the oxidative damage induced by hydrogen peroxide [54]. This flavonol helps to prevent oxidative damage to cells and DNA. No genotoxic effect of AECal on the model used has been detected. Therefore, the results obtained encourage studies on its pharmacological properties.

## 5. Conclusion

From these results, it is tempting to speculate that the primary mechanism of CF-AECal-induced bronchodilatation results from interference with membrane polarisation through either direct or indirect BK channels activation. By shifting the CCRC without affecting the efficacy of acetylcholine, CF-AECal at low-level concentrations may therefore play a role of cholinergic muscarinic receptors inhibitor. However, further studies are required to clarify these speculations and determine the precise mechanism and molecules by which *Cassia alata* extracts produce relaxation of tracheal smooth muscles. Evaluation of isolated bioactive molecules from plant extract eliciting tracheorelaxant effect may give new investigational and treatment tools in bronchorespiratory pharmacology. This study provides sound mechanistic basis for the use of *Cassia alata* in asthma-induced bronchospasm. The negative data obtained in the present study on the genotoxic potential of the tested compounds encourage the development of research on other genotoxic endpoints in screening studies for *Cassia alata* extract safety. In the antigenotoxicity area in particular, it is worth investigating the chemopreventive activity of *Cassia alata* extracts against various DNA damaging agents.

## Figures and Tables

**Table 1:** Pharmacodynamic parameters (% relaxation at 1 mg/mL, EC_50_ values, and potency (pEC_50_)) and SEM obtained by nonlinear regression analysis of concentration-response curve for AECal, CF-AECal, EAF-AECal, and nBF-AECal in isolated rat trachea precontracted by acetylcholine (10 *µ*M).

	AECal	PEF-AECal	CF-AECal	EAF-AECal	nBF-AECal
Contraction (% of max)	5.37 ± 1.37	98.79 ± 3.52	−9.67 ± 2.60	94.27 ± 5.69	98.47 ± 4.03
EC_50_ (CI)	0.54 (0.30–0.98)	nd	0.02 (0.02–0.03)	nd	nd
pEC_50_ ± SEM	0.27 ± 0.12	nd	1.60 ± 0.06	−1.47 ± 0.25	nd

Data are expressed as the mean ± SEM of 6–8 experiments. Significantly different from control: *P < 0.05, **P < 0.01, and ***P < 0.001.

**Table 2:** Pharmacodynamic parameters (% relaxation at 1 mg/mL, EC_50_ values, and potency (pEC_50_)) and SEM obtained by nonlinear regression analysis of concentration-response curve for AECal, in isolated rat trachea precontracted by acetylcholine (10 *µ*M) and KCl (30 mM, 80 mM).

Contractant	Contraction (% of max)	EC_50_ (95% CI)	pEC_50_ ± SEM
Ach (10^−5^ M)	−9.67 ± 2.60	0.024 (0.019–0.031)	1.62 ± 0.07
KCl (30 mM)	29.48 ± 6.04	0.05 (0.027–0.100)	1.29 ± 0.15
KCl (80 mM)	55.47 ± 7.42	0.49 (0.151–1.596)***	−0.35 ± 0.60***

Data are expressed as the mean ± SEM of 6–8 experiments. Significantly different from control: *P < 0.05, **P < 0.01, and ***P < 0.001.

**Table 3:** *E*_max⁡_, EC_50 _values, and potency (pEC_50_) of acetylcholine under various experimental conditions. CF-AECal concentrations are expressed in mg/mL.

	ACh	ACh		
		CF-AECal 0.03	CF- AECal 0.1	CF-AECal 1
*E*_max⁡_ ± SEM	95.22 ± 2.55	94.44 ± 3.004	98.09 ± 2.92	43.63 ± 2.29***
pEC_50_ ± SEM	4.95 ± 0.07	4.60 ± 0.08	4.11 ± 0.07	3.32 ± 0.102

Data are expressed as the mean ± SEM of 6–8 experiments. Significantly different from control: *P < 0.05, **P < 0.01, and ***P < 0.001.

**Table 4:** EC_50 _values and potency (pEC_50_) of CF-AECal-induced relaxation of rat tracheal smooth muscle contracted by 10 *µ*M acetylcholine under various experimental conditions.

Treatment groups	EC_50_ (mg/mL)	pEC_50_
Control	0.021 (0.013–0.031)	1.68 ± 0.08
L-NAME (100 *µ*M)	0.018 (0.010–0.031)	1.4 ± 0.09
Indomethacin (10 *µ*M)	0.019 (0.011–0.031)	1.73 ± 0.11
Methylene blue (100 *µ*M)	0.021 (0.008–0.054)	1.68 ± 0.15
Propranolol (1 *µ*M)	0.029 (0.010–0.081)	1.54 ± 0.16
TEA (5 mM)	0.184 (0.123–0.274)	0.74 ± 0.08***
Glibenclamide (10 *µ*M)	0.022 (0.015–0.032)	1.65 ± 0.07

EC_50_ was obtained from the concentration-response curve of CF-AECal. Data are expressed as the mean ± SEM of 6–8 experiments. Significantly different from control: *P < 0.05, **P < 0.01, and ***P < 0.001.

## Abbreviations

AECal: Aqueous-ethanolic extract from *Cassia alata *
PEF-AECal: Petroleum ether fraction from AECal
CF-AECal: Chloroformic fraction from AECal
EAF-AECal: Ethyl acetate fraction from AECal
nBF-AECal: n-Butanol fraction from AECal
CP: Cyclophosphamide.
